# Genetic susceptibility and clinical validation of ICAM-5 in immune-mediated non-infectious uveitis

**DOI:** 10.3389/fimmu.2026.1948697

**Published:** 2026-10-06

**Authors:** Zihao Zhuang, Pengcheng Hu, Xuanming Hong, Tianfeng Lin, Yang Yu, Xinyu Jia, Xuemei Su, Binling Su, Wenjian Shi, Jianmin Hu

**Affiliations:** 1 Department of Ophthalmology, The Second Affiliated Hospital of Fujian Medical University, Quanzhou, Fujian, China; 2 Engineering Research Center of Assistive Technology for Visual Impairment, Fujian Provincial Universities, Quanzhou, Fujian, China; 3 Department of Epidemiology and Biostatistics, School of Public Health, Peking University, Beijing, China; 4 Department of Plastic Surgery, The Second Affiliated Hospital of Fujian Medical University, Quanzhou, Fujian, China

**Keywords:** circulating biomarker, ICAM-5, inflammation, Mendelian randomization, multi-omics, noninfectious uveitis

## Abstract

**Purpose:**

To investigate genetically supported molecular factors associated with uveitis and to evaluate the clinical, transcriptomic, and retinal relevance of intercellular adhesion molecule 5 (ICAM-5) across major immune-mediated non-infectious uveitis subtypes.

**Methods:**

Two-sample Mendelian randomization (MR) was conducted using eQTLGen eQTL data and two independent pQTL resources (Icelandic SomaScan and FinnGen R10), with uveitis GWAS summary statistics as the outcome. *ICAM5* was evaluated using complementary MR estimators, sensitivity analyses, reverse MR, and Bayesian colocalization. Plasma ICAM-5 concentrations were measured by ELISA in 90 patients with active non-infectious uveitis (30 each with Vogt-Koyanagi-Harada disease, Behçet’s disease-associated uveitis, and HLA-B27-associated acute anterior uveitis) and 40 healthy controls using age- and sex-adjusted models. *ICAM5* expression was further examined in the peripheral-blood transcriptomic dataset GSE194060, whereas retinal *Icam5* expression and cellular distribution were evaluated using experimental autoimmune uveitis retinal single-cell RNA-sequencing data and GSE132229.

**Results:**

Genetically predicted higher *ICAM5* expression and ICAM-5 protein levels were associated with increased uveitis susceptibility in the eQTLGen dataset (OR = 2.46, 95% CI 1.48–4.11, *P* = 5.69 × 10^-4^; nSNP =8), the Icelandic SomaScan cis-pQTL analysis (OR = 1.27, 95% CI 1.11–1.46, *P* = 5.30 × 10^-4^; nSNP =89), and the FinnGen R10 cis-pQTL analysis (OR = 1.21, 95% CI 1.07–1.38, *P* = 3.13 × 10^-^³; nSNP =4). Sensitivity analyses showed no significant heterogeneity or horizontal pleiotropy, and reverse MR provided no evidence of an association from uveitis liability to ICAM-5 levels. Plasma ICAM-5 concentrations were higher in all three uveitis subgroups than in healthy controls and remained elevated after age and sex adjustment, with the greatest increase in Vogt-Koyanagi-Harada disease. In the GSE194060 peripheral-blood transcriptomic dataset, *ICAM5* expression was higher in the uveitis group. Analysis of the retinal single-cell RNA-sequencing data showed that *Icam5* was predominantly detected in neuronal populations.

**Conclusion:**

Multi-omics MR, clinical protein quantification, transcriptomic analysis, and retinal single-cell profiling provide convergent evidence linking ICAM-5 to immune-mediated non-infectious uveitis. These findings support ICAM-5 as a genetically supported candidate mediator and circulating biomarker.

## 1 Introduction

Uveitis comprises a heterogeneous group of intraocular inflammatory disorders that can damage the uveal tract, retina, and optic nerve (1, 2). It accounts for approximately 10%-15% of blindness worldwide and disproportionately affects people of working age, imposing a substantial burden on public health and quality of life (3). Immune-mediated non-infectious uveitis includes clinically distinct subtypes with different inflammatory profiles, tissue involvement, and treatment responses. Current management relies largely on corticosteroids and immunosuppressive agents, but broad immunomodulation may be limited by systemic toxicity and incomplete disease specificity (4). Defining molecular factors associated with disease susceptibility and activity is therefore important for improving biological stratification and guiding the development of more targeted approaches. Genetic causal-inference studies have also linked systemic immune-mediated disorders with ocular inflammatory phenotypes, including iridocyclitis (5).

Genome-wide association studies (GWAS) have identified genetic loci associated with uveitis, but linking these signals to specific genes, proteins, and disease-relevant pathways remains challenging (6). Observational molecular measurements can be affected by confounding and reverse causation, and a single genetic signal may influence multiple molecular traits or pathways. Integrating expression quantitative trait locus (eQTL) and protein quantitative trait locus (pQTL) resources with two-sample Mendelian randomization (MR) provides a framework for evaluating the association between genetically predicted molecular traits and disease susceptibility (6, 7). Recent studies have extended MR to corneal disease and glaucoma (8–11). By utilizing genetic variants as unconfounded instrumental variables, MR allows for the inference of causal relationships between molecular exposures (gene or protein expression) and clinical outcomes. This approach effectively mimics randomized controlled trials in silico, prioritizing drug targets with a higher probability of success in subsequent clinical development (12, 13). Complementary analyses of instrument strength, heterogeneity, horizontal pleiotropy, reverse direction, and locus-level colocalization can further assess the consistency and genomic context of these associations. This framework can therefore prioritize candidates for subsequent clinical and biological evaluation. Related approaches combining circulating pQTLs, MR, and colocalization have also been used to prioritize molecular targets in ocular disease (14).

We therefore prioritized *ICAM5*, the only candidate represented across all three QTL resources with nominal associations in concordant directions, for focused genetic, clinical, transcriptomic, and retinal evaluation. The study aimed to assess whether ICAM-5 represents a genetically supported candidate mediator and circulating biomarker of immune-mediated non-infectious uveitis.

## 2 Methods

### 2.1 Study design

To investigate genetically supported molecular factors associated with uveitis and assess their clinical and biological relevance, we established an integrated framework spanning multi-omics screening, genetic evaluation, clinical validation, and experimental assessment. Genome-scale eQTL data from eQTLGen and pQTL data from Icelandic SomaScan and FinnGen R10 were used as exposures, with uveitis GWAS summary statistics as the outcome. Following instrumental-variable selection and harmonization, two-sample MR was used to screen candidate traits. Cross-dataset comparison prioritized *ICAM5* for focused analysis. Its genetic association with uveitis was further characterized using complementary MR estimators, instrument-strength assessment, heterogeneity and horizontal-pleiotropy tests, MR-PRESSO, reverse MR, and Bayesian colocalization.

Clinical validation was conducted in an independent cohort of 90 patients with active immune-mediated non-infectious uveitis and 40 healthy controls. The patient cohort comprised Vogt–Koyanagi–Harada disease (n =30), Behçet disease-associated uveitis (n =30), and HLA-B27-associated acute anterior uveitis (n =30). Plasma ICAM-5 concentrations were quantified by ELISA, compared between patients and controls, and evaluated across disease subtypes using age- and sex-adjusted models.

To examine the transcriptomic and ocular context of *ICAM5*, expression was assessed in the human non-infectious uveitis dataset GSE194060. Retinal expression was further evaluated by single-cell RNA sequencing in experimental autoimmune uveitis mice, while GSE132229 was analyzed to define the cellular distribution of *Icam5* across retinal populations and its expression pattern under inflammatory conditions. These sequential analyses linked multi-omics prioritization with human plasma measurements, public transcriptomic evidence, and retinal single-cell validation. The overall workflow is shown in **Figure 1**.

**Figure 1:**
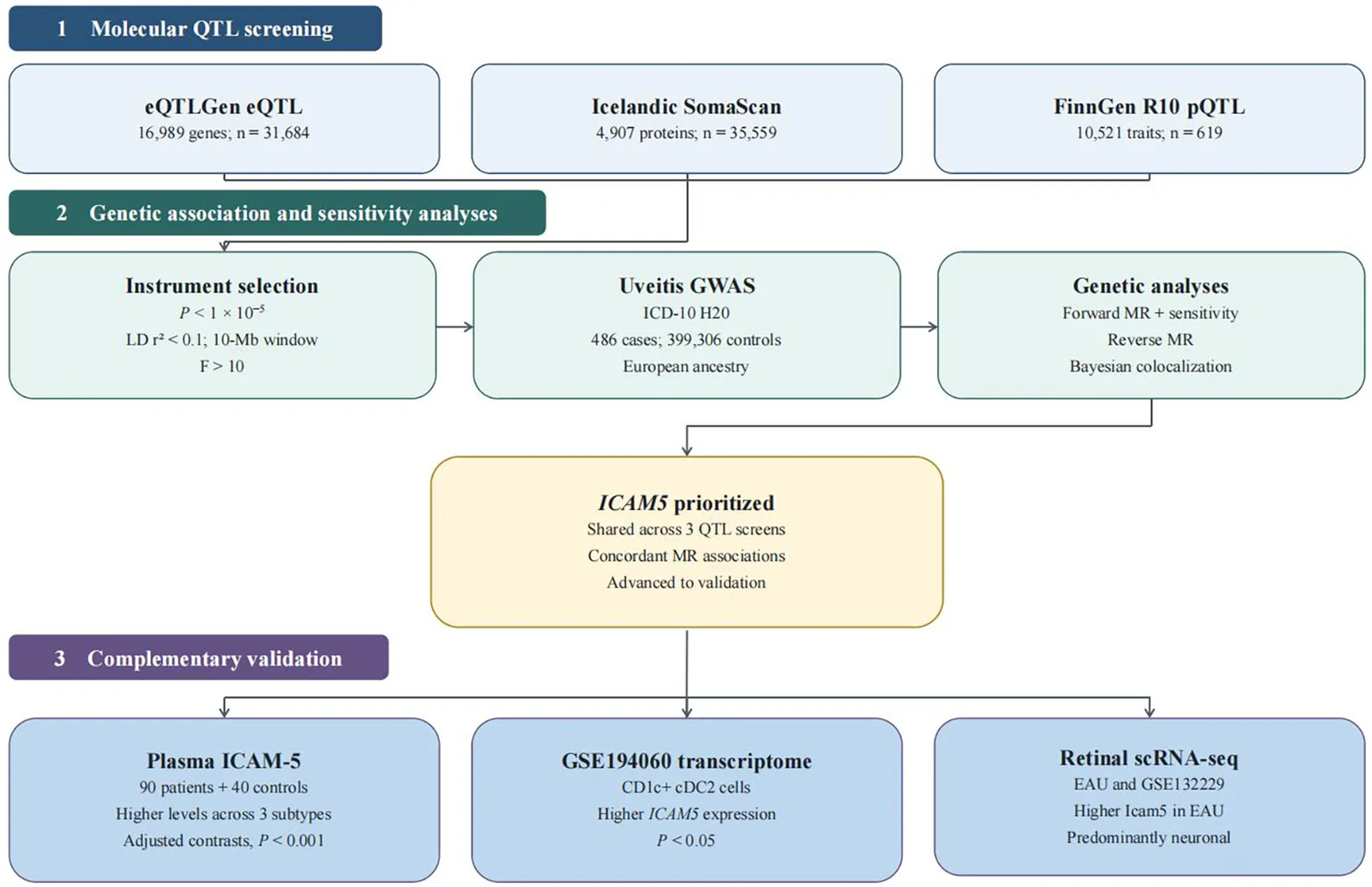
Overview of the study design and analytical framework.

### 2.2 Exposure and outcome data sources

To comprehensively capture genetic variations regulating gene and protein expression, three independent multi-omics datasets were utilized as exposure sources:

The eQTL data for this study were sourced from the eQTLGen Consortium. This dataset encompassed 16,989 genes derived from blood transcriptomic profiles of 31,684 healthy individuals of European ancestry (15).

Two independent pQTL resources were used to evaluate genetically predicted plasma protein abundance. The Icelandic SomaScan study measured 4,907 plasma proteins in 35,559 individuals and identified 18,084 significant protein-variant associations (16). The FinnGen R10 pQTL resource comprised 10,521 protein traits based on 619 samples and provided genetic variants associated with plasma protein levels (17).

GWAS summary statistics for uveitis were obtained from Zhou et al. (18). The dataset included 399,792 individuals of European ancestry from the United Kingdom, comprising 486 cases and 399,306 controls. Uveitis cases were defined using the International Classification of Diseases, 10th Revision (ICD-10) code H20. More than 24 million single-nucleotide polymorphisms were evaluated in the GWAS.

### 2.3 Instrumental variable selection

QTL-associated single-nucleotide polymorphisms (SNPs) meeting a prespecified suggestive association threshold of *P* < 1×10–^5^ were selected as candidate instruments (9, 19). Linkage disequilibrium (LD) clumping was performed in PLINK using the 1000 Genomes European reference panel, with r^2^ < 0.1 within a 10,000-kb window (20–22). Effect alleles were harmonized between the exposure and outcome datasets. Variants with incompatible alleles or unresolved strand orientation were excluded; palindromic variants were retained only when allele orientation could be resolved using allele-frequency information. Candidate instruments were screened in the GWAS Catalog for associations with rheumatoid arthritis, inflammatory bowel disease, multiple sclerosis, or systemic lupus erythematosus. Variants with these associations were excluded to reduce potential confounding and horizontal pleiotropy (23). Instrument strength was assessed using the F statistic. Only instruments with F > 10 were retained. All genomic coordinates used for *ICAM5* instrument classification were harmonized to GRCh38. The *ICAM5* locus was defined as chr19:10,289,952-10,296,778, and cis-pQTLs were prespecified as variants located on chromosome 19 within ±1 Mb of this interval (chr19:9,289,952-11,296,778). Variants outside this region or on another chromosome were classified as trans-pQTLs. In the FinnGen R10 resource, rs507666 and rs9411492 mapped to chromosome 9 and were therefore classified as trans-pQTLs and excluded from the primary target-specific MR analysis. The primary FinnGen analysis was restricted to the four eligible cis-pQTL instruments after LD clumping and harmonization.

### 2.4 Mendelian randomization analysis

Mendelian randomization (MR) analyses were conducted using the “TwoSampleMR” R package (version 0.5.7) (24). To estimate the causal effects of genes instrumented by multiple SNPs, SNP-specific Wald ratios were meta-analyzed using the inverse-variance weighted (IVW), weighted median, MR-Egger methods, simple mode and weighted mode. Specifically, the IVW approach was adopted as the primary estimator due to its superior statistical efficiency under the assumption that all genetic instruments are valid (25, 26). In parallel, the weighted median method was utilized as a robust alternative to provide consistent estimates even when up to 50% of the instruments were invalid. Furthermore, MR-Egger regression was applied to detect and correct for potential directional pleiotropy, albeit with reduced statistical power (27). Consistency of results across these methods was considered evidence that pleiotropy did not substantially bias the causal estimates. To further assess heterogeneity and directional pleiotropy for genes with more than two instruments, Cochran’s Q statistic and the MR-Egger intercept test were applied, respectively (28). In cases of discordance among the MR methods, the IVW results were prioritized. Ultimately, this multi-stage analytical strategy was established to minimize false positives and enhance the external validity of the findings.

### 2.5 Colocalization analysis

Bayesian colocalization analysis was performed for *ICAM5* using the “coloc” R package (version 5.2.3) to assess whether the MR associations were likely driven by a shared causal variant rather than LD between distinct signals. For each *ICAM5* eQTL and pQTL signal, variants within the *ICAM5* cis-region were extracted from the corresponding QTL summary statistics and harmonized with the uveitis GWAS data. Shared variants were retained after allele harmonization, and ambiguous palindromic variants were excluded. Evidence for colocalization was defined as a posterior probability for a shared causal variant (PP.H4) ≥0.70.

### 2.6 Reverse causality detection

To investigate potential reverse causation, a bidirectional MR analysis was conducted (29). Specifically, genetic instruments for uveitis were selected from the FinnGen dataset, employing screening criteria consistent with those applied for eQTL analysis. Causal effect estimates were derived via the inverse-variance weighted, weighted median, and MR-Egger methods, where associations with a p-value <0.05 were considered statistically significant. Furthermore, the directionality of the putative causal relationship between pQTLs and uveitis was further validated through Steiger filtering.

### 2.7 Clinical cohort and plasma ICAM-5 measurement

To clinically corroborate the genetic findings derived from the Mendelian randomization analysis, a case-control study was conducted to validate ICAM-5 at the protein level. A total of 90 patients with immune-mediated non-infectious uveitis and 40 healthy controls, all of Han Chinese ethnicity, were enrolled. To address the etiological heterogeneity of uveitis, the patient cohort was stratified into three well-defined subgroups (n =30 per group): Vogt-Koyanagi-Harada (VKH) disease (diagnosed according to the international criteria) (30, 31), Behçet’s disease-associated uveitis (BDU, confirmed by the International Study Group for Behçet’s Disease criteria with documented ocular involvement) (32, 33), and HLA-B27-associated acute anterior uveitis (HLA-B27^+^ AU; defined by a typical acute anterior uveitis phenotype with positive HLA-B27 antigen and exclusion of other identifiable etiologies) (34, 35). All enrolled patients presented with active disease at the time of sampling. Strict exclusion criteria were applied to the patient group to rule out infectious uveitis, secondary inflammation following intraocular surgery or ocular trauma, concomitant systemic autoimmune disorders, malignancies, severe cardiovascular diseases, and diabetes mellitus. The healthy control group consisted of individuals undergoing routine physical examinations and clinically recruited healthy volunteers. These individuals were thoroughly screened to ensure the absence of any ocular diseases, immune-related disorders, malignancies, cardiovascular and metabolic disorders, or active systemic infections. Patients were eligible if they were first-onset cases or had discontinued systemic corticosteroids, immunosuppressants, and biologic agents for at least 1 year before sampling. During the current episode, only topical corticosteroid eye drops were used.

Venous blood samples (5 mL) were obtained from the antecubital vein of each participant. Plasma was isolated via centrifugation within 2 hours of collection and stored at -80 °C until further analysis. Plasma ICAM-5 concentrations were quantified using a sandwich ELISA kit (ADS-0420H1, Human *ICAM5* ELISA Kit; Jiangsu Aidisheng Biological Technology Co., Ltd., China) strictly adhering to the manufacturer’s protocols.

Plasma ICAM-5 concentrations were summarized as median (interquartile range, IQR) because of their non-normal distribution. Individual values were displayed with box plots. Unadjusted differences across the four groups were evaluated using the Kruskal-Wallis H test, followed by Mann-Whitney U tests with Bonferroni correction for *post-hoc* pairwise comparisons. To assess whether group differences were independent of demographic imbalance, we fitted multivariable linear regression models. Natural-log-transformed plasma ICAM-5 concentration was the outcome, and disease group, age (per 10-year increment), and sex were covariates. Healthy controls served as the reference group. Heteroscedasticity-consistent (HC3) standard errors were used. Disease-versus-control associations were reported as adjusted geometric mean ratios (GMRs) with 95% confidence intervals. *P* values for the three prespecified disease-versus-control contrasts were adjusted using the Holm method. As systemic treatment did not vary across eligible cases, it was treated as an eligibility criterion rather than a model covariate. All tests were two-sided, and *P* < 0.05 was considered statistically significant.

### 2.8 Transcriptomic validation in a public non-infectious uveitis dataset

To corroborate *ICAM5* dysregulation at the transcriptomic level, gene-expression data and sample annotations for the non-infectious uveitis (NIU) dataset GSE194060 were retrieved from the Gene Expression Omnibus (GEO) database. This dataset includes RNA-sequencing profiles of CD1c^+^ conventional type 2 dendritic cells purified from peripheral blood mononuclear cells of 28 patients with NIU and 14 healthy controls. Gene-level expression values were normalized to transcripts per million (TPM) and log_2_(TPM + 1)-transformed. *ICAM5* expression was compared between the NIU and healthy-control groups using a two-sided Welch’s two-sample t-test; *P* < 0.05 was considered nominally significant. Expression distributions were visualized as box-and-jitter plots with overlaid individual data points using the R package ggplot2.

### 2.9 Experimental autoimmune uveitis model and retinal single-cell RNA sequencing

Six female C57BL/6J mice (6–8 weeks old; specific-pathogen-free; Hunan Silaikejingda Laboratory Animal Co., Ltd.) were maintained under a 12-h light/dark cycle with food and water available ad libitum and randomly assigned to normal control (NC, n =3) or experimental autoimmune uveitis (EAU, n =3) groups. EAU was induced as described previously (36, 37). Briefly, 500 μg human IRBP651–670 was dissolved in PBS containing 20% DMSO and emulsified 1:1 with complete Freund’s adjuvant containing 5 mg/mL heat-killed Mycobacterium tuberculosis. Under 1.25% tribromoethanol anesthesia (0.2 mL/10 g body weight), each mouse received 200 μL of emulsion by subcutaneous injection at multiple sites, followed by 1 μg pertussis toxin in 100 μL PBS intraperitoneally. On day 14 after immunization, mice were euthanized and bilateral retinas from each mouse were pooled as one biological sample.

Retinas were dissociated using the SeekMate Tissue Dissociation Kit A Pro (SeekGene, K01801301). After erythrocyte lysis, cell viability was assessed by AO/PI staining, and samples with viability >80% were used for library preparation. Single-cell 3’ mRNA libraries were generated using the SeekOne Digital Droplet Single Cell 3’ Library Preparation Kit (SeekGene, K00202) and sequenced on an Illumina NovaSeq 6000 platform (paired-end 150 bp). Raw reads were processed using SeekSoul Tools, aligned to the mouse reference genome with STAR, and quantified with featureCounts to generate cell-by-gene matrices. Downstream analyses used Seurat (v4.1.1): cells with >20% mitochondrial transcripts, >5% hemoglobin transcripts, or <300 or >6,000 detected genes were excluded; doublets were removed with DoubletFinder; batch effects were corrected with Harmony; and the top 2,000 variable genes and first 20 principal components were used for clustering and dimensionality reduction (38–40). *Icam5* expression was compared between groups using a two-sided Wilcoxon rank-sum test at the cell level. The GSE132229 dataset was analyzed through its published online portal to examine *Icam5* expression across retinal cell subsets in wild-type and *Aire-knockout* mice (41).

## 3 Results

### 3.1 Molecular-trait screening and instrument characteristics

Across the eQTLGen, Icelandic SomaScan, and FinnGen R10 resources, 16,989 gene-expression traits, 4,907 plasma proteins, and 10,521 protein traits, respectively, were evaluated. Screening at the prespecified threshold yielded 1,693 eQTLGen candidates, 262 unique Icelandic candidates from 267 candidate-list rows, and 26 FinnGen candidates. Their union comprised 1,938 unique candidates, and *ICAM5* was the only candidate represented in all three screens (**Supplementary Figure 1**; **Supplementary Tables 1**, **2**). Although *ICAM5* did not meet the prespecified FDR significance criterion in the genome-scale screening, it showed nominally significant and directionally consistent associations across the eQTLGen, Icelandic SomaScan, and FinnGen R10 resources. Given this cross-resource consistency, *ICAM5* was retained for focused downstream analyses, including instrument-level assessment, sensitivity analyses, colocalization, reverse MR, and clinical and transcriptomic validation. The screening scope and corresponding multiple-testing results are provided in **Supplementary Table 1**.

The primary *ICAM5* instrument sets comprised 8 eQTLGen SNPs, 89 Icelandic SomaScan cis-pQTL SNPs, and 4 FinnGen R10 cis-pQTL SNPs; 92 Icelandic SomaScan trans-pQTL SNPs were excluded from the primary analysis. Two additional FinnGen variants, rs507666 and rs9411492, were located on chromosome 9 and were therefore classified as trans-pQTLs and excluded from the primary cis-restricted analysis. Detailed instrument characteristics and single-SNP estimates are provided in **Supplementary Tables 3**-**5**. All retained primary instruments showed adequate strength, with F statistics >10.

### 3.2 Genetically predicted association between *ICAM5* and uveitis susceptibility

IVW analyses showed positive associations of genetically predicted *ICAM5* expression or circulating ICAM-5 abundance with uveitis susceptibility in all three datasets. The eQTLGen estimate was OR = 2.46 (95% CI, 1.48-4.11; *P* = 5.69×10^-4^; nSNP = 8). The Icelandic SomaScan cis-pQTL and FinnGen R10 cis-pQTL estimates were OR = 1.27 (95% CI, 1.11-1.46; *P* = 5.30×10^-4^; nSNP =89) and OR = 1.21 (95% CI, 1.07-1.38; *P* = 3.13×10^-3^; nSNP =4), respectively (**Figure 2**). Effect estimates from all five MR estimators were positive in each dataset, although statistical support varied among estimators (**Supplementary Table 6**; **Supplementary Figure 2**).

**Figure 2:**
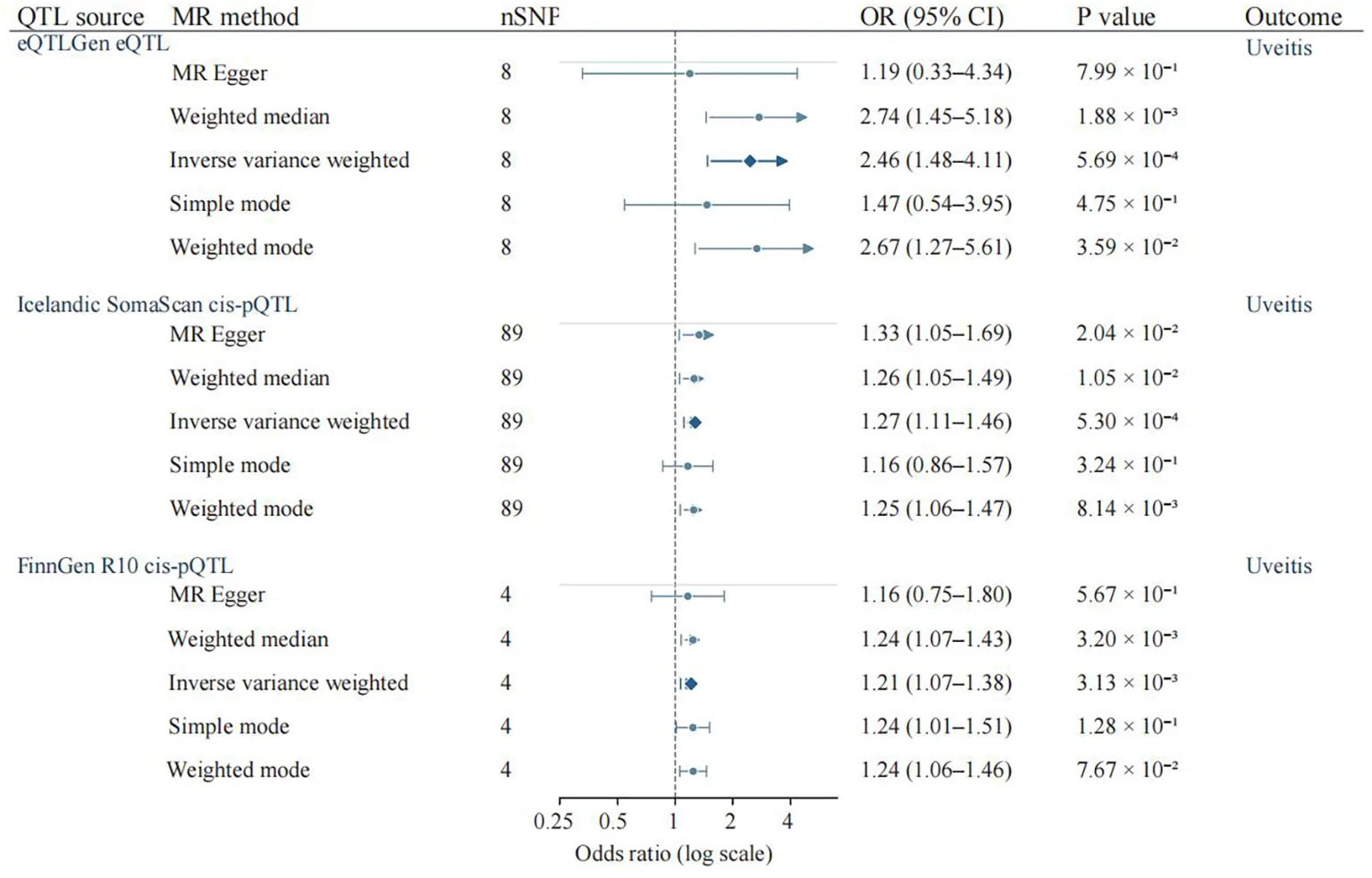
Forest plots of forward Mendelian randomization estimates for the association of genetically predicted *ICAM5* expression or ICAM-5 protein abundance with uveitis susceptibility. MR, Mendelian randomization; SNP, single nucleotide polymorphism; CI, confidence interval.

The IVW Cochran’s Q test *P* values were 0.656, 0.103, and 0.442 for eQTLGen, Icelandic SomaScan, and FinnGen R10, respectively (**Supplementary Table 7**). The corresponding MR-Egger intercept *P* values were 0.275, 0.646, and 0.290 (**Supplementary Table 8**), and the MR-PRESSO global *P* values were 0.730, 0.054, and 0.459 (**Supplementary Table 9**). Reverse MR included 11 instruments for the Icelandic outcome and 10 for the Olink outcome, with a minimum F statistic of 20.87 in both analyses. The IVW estimates were OR = 1.000 (95% CI, 0.987-1.013; *P* = 0.964) and OR = 0.967 (95% CI, 0.873-1.070; *P* = 0.513), respectively (**Supplementary Table 10**; **Supplementary Figure 3**). For the evaluated colocalization comparisons, PP.H4 was below the prespecified threshold of 0.70.

### 3.3 Clinical validation of plasma ICAM-5 levels

For clinical validation, we enrolled 90 patients with active immune-mediated non-infectious uveitis and 40 healthy controls (HC). The uveitis cohort comprised 30 patients each with Vogt-Koyanagi-Harada (VKH) disease, Behçet’s disease-associated uveitis (BDU), and HLA-B27-associated acute anterior uveitis (HLA-B27^+^ AU). All patients were first-onset cases or had discontinued systemic corticosteroids, immunosuppressants, and biologic agents for at least 1 year before sampling; only topical corticosteroid eye drops were used during the current episode. Sex distribution did not differ among groups (Pearson’s χ2 = 2.24, df =3, P = 0.523), whereas age differed among groups (Welch’s one-way analysis of variance, P = 0.0017; **Table 1**).

**Table 1:** Demographic characteristics of study participants across groups.

Group	n	Male, n (%)	Female, n (%)	Age, years (Mean ± SD)	Age range, years (Min–Max)
HC	40	22 (55.0)	18 (45.0)	31.03 ± 9.29	18–55
VKH	30	18 (60.0)	12 (40.0)	41.73 ± 15.86	16–71
BDU	30	18 (60.0)	12 (40.0)	36.97 ± 10.04	21–60
HLA-B27^+^ AU	30	13 (43.3)	17 (56.7)	38.67 ± 10.56	20–57

Values are n (%) or mean ± SD, unless otherwise indicated. Sex distribution was compared using Pearson’s chi-square test (*P* = 0.523). Age was compared using Welch’s one-way analysis of variance (*P* = 0.0017).

HC, healthy controls; VKH, Vogt-Koyanagi-Harada disease; BDU, Behçet’s disease-associated uveitis; HLA-B27^+^ AU, HLA-B27–associated acute anterior uveitis.

Median plasma ICAM-5 concentrations were 0.079 ng/mL (IQR, 0.037-0.120) in HC, 1.281 ng/mL (IQR, 0.876-2.041) in VKH, 0.375 ng/mL (IQR, 0.265-0.545) in BDU, and 0.455 ng/mL (IQR, 0.283-0.601) in HLA-B27^+^ AU. The overall group difference was significant (Kruskal-Wallis H test, P < 0.001; **Figure 3a**). Each uveitis subgroup had higher concentrations than HC in Bonferroni-adjusted pairwise comparisons (all P < 0.001). VKH also had higher concentrations than BDU and HLA-B27^+^ AU (both P < 0.001), whereas BDU and HLA-B27^+^ AU did not differ (P > 0.05).

**Figure 3:**
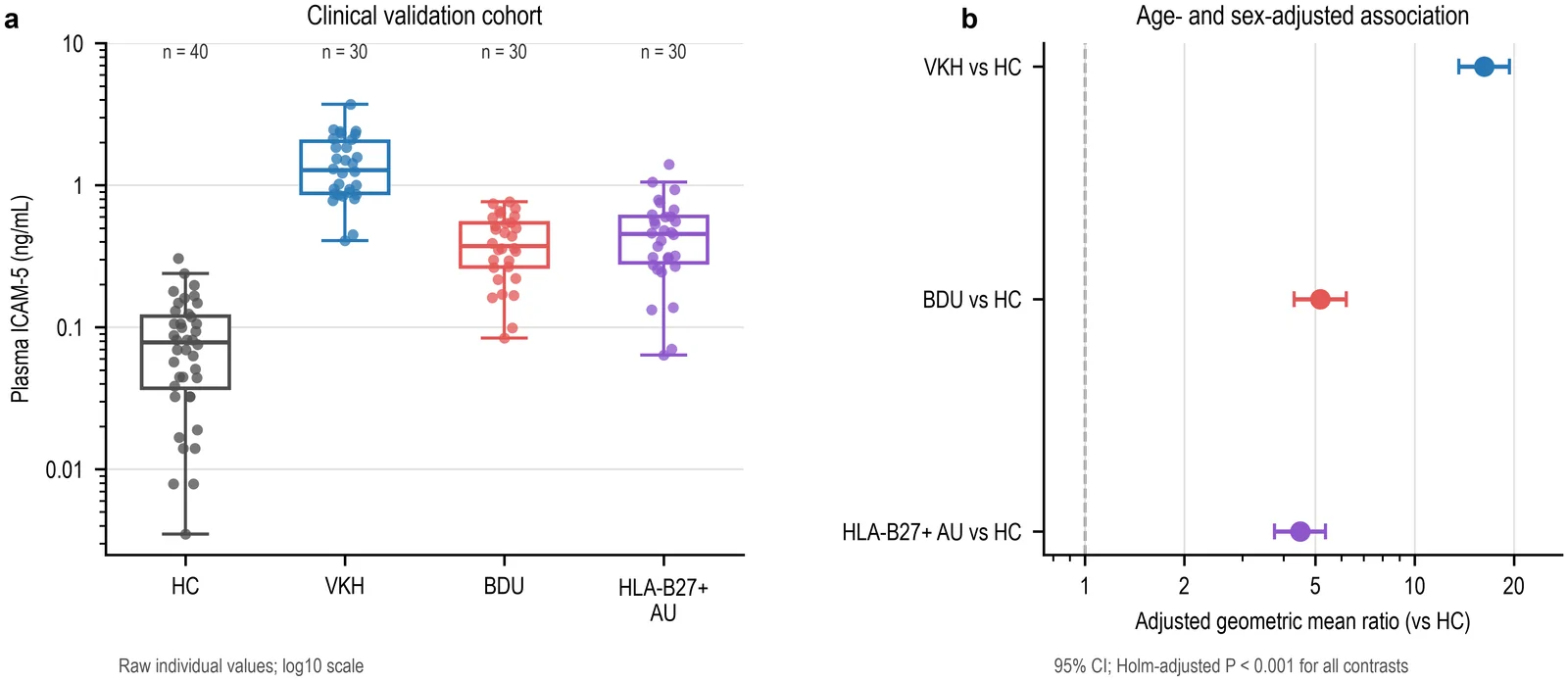
Plasma ICAM-5 concentrations and age- and sex-adjusted associations in the clinical validation cohort. **(a)** Individual plasma ICAM-5 concentrations are shown for 40 healthy controls (HC), 30 patients with VKH, 30 with BDU, and 30 with HLA-B27^+^ AU. Boxes indicate the median and interquartile range, and whiskers indicate 1.5 × the interquartile range. The y-axis is log10-scaled. **(b)** Adjusted GMRs and 95% confidence intervals were estimated using a multivariable linear model of log-transformed plasma ICAM-5 concentration, with disease group, age, and sex as covariates. HC was the reference group. P values were Holm-adjusted across the three prespecified disease-versus-HC contrasts. All adjusted contrasts had P < 0.001. ICAM-5, intercellular adhesion molecule-5; VKH, Vogt-Koyanagi-Harada disease; BDU, Behçet’s disease-associated uveitis; HLA-B27^+^ AU, HLA-B27–associated acute anterior uveitis; HC, healthy controls.

After adjustment for age and sex, each uveitis subgroup remained associated with higher plasma ICAM-5 concentrations than HC (**Figure 3b**). The adjusted geometric mean ratio was 16.25 (95% CI, 13.61-19.40) for VKH, 5.17 (95% CI, 4.31-6.20) for BDU, and 4.49 (95% CI, 3.76-5.36) for HLA-B27^+^ AU (all Holm-adjusted P < 0.001). The model included all 130 participants and had an R^2^ of 0.930; complete model estimates are provided in **Supplementary Table 11** and **Supplementary Figure 4**.

### 3.4 *ICAM5* expression in GSE194060

The GSE194060 analysis included CD1c^+^ conventional type 2 dendritic cells from 28 patients with non-infectious uveitis and 14 healthy controls. After TPM normalization and log2(TPM + 1) transformation, *ICAM5* expression was higher in the uveitis group (two-sided Welch’s t-test, P < 0.05; **Supplementary Figure 5**).

### 3.5 Retinal *Icam5* expression

Retinal single-cell RNA sequencing was performed on three normal-control and three EAU biological samples collected on day 14 after immunization. At the cell level, scaled *Icam5* expression was higher in EAU retinal cells (two-sided Wilcoxon rank-sum test, P = 0.026; **Supplementary Figure 6**). In GSE132229, *Icam5* expression was concentrated in retinal neuronal populations, principally amacrine cells and retinal ganglion cells, and was negligible in the displayed intraretinal immune-cell subsets. Mean normalized *Icam5* expression in retinal ganglion cells was higher in *Aire-knockout* than in wild-type retinas (**Supplementary Figure 7**).

## 4 Discussion

The present study integrated multi-omics MR analysis with clinical cohort validation to investigate the genetically supported association between intercellular adhesion molecule-5 (ICAM-5, also known as telencephalin) and uveitis, with particular emphasis on immune-mediated non-infectious subtypes. By using genetic variants as instrumental variables to reduce bias from conventional confounding and reverse causation (25), the primary IVW analysis showed that genetically predicted higher *ICAM5* expression and ICAM-5 protein levels were consistently associated with increased uveitis susceptibility across the evaluated molecular datasets, including the cis-restricted Icelandic SomaScan and FinnGen analyses. Sensitivity analyses using weighted median, MR-Egger, simple mode, and weighted mode methods generally showed concordant directions, although the precision of the estimates varied among methods. In the reverse MR analysis, uveitis susceptibility was not significantly associated with circulating ICAM-5 levels, a finding compatible with the inferred direction of association (7).

In the GWAS dataset, the uveitis outcome was defined according to the ICD-10 H20 coding category, which primarily captures iridocyclitis and anterior uveitis phenotypes. In population-based genetic studies, this coding category may capture a phenotype enriched for non-infectious uveitis, although it does not fully resolve etiological heterogeneity. Pathogen-defined infectious ocular inflammation is more likely to be recorded under infection-specific diagnostic codes, with ocular involvement additionally captured by corresponding ophthalmic codes when applicable. To place the population-level genetic findings in more clinically defined disease contexts, we performed multidimensional validation in three common and well-characterized non-infectious uveitis subtypes: Vogt-Koyanagi-Harada (VKH) disease, Behçet’s disease-associated uveitis (BDU), and HLA-B27-associated acute anterior uveitis (HLA-B27+ AU). All clinical cases were diagnosed according to established international criteria (32–35), allowing a more stringent assessment of ICAM-5 dysregulation across defined inflammatory phenotypes.

At the protein level, enzyme-linked immunosorbent assay (ELISA) validation in an independent clinical cohort showed substantially higher plasma ICAM-5 concentrations in patients with immune-mediated non-infectious uveitis than in healthy controls. Subgroup analysis demonstrated elevated concentrations in VKH, BDU, and HLA-B27^+^ AU, with the most pronounced increase observed in the VKH subgroup. In parallel, the public GSE194060 dataset showed nominally higher *ICAM5* in CD1c^+^ conventional type 2 dendritic cells from patients with non-infectious uveitis (42) (**Supplementary Figure 5**). Although this transcriptomic signal does not establish the cellular source of circulating ICAM-5, it provides independent molecular evidence that is directionally consistent with the plasma protein findings. Together, the genetic, transcriptomic, and protein-level results provide convergent support for an association between ICAM-5 dysregulation and immune-mediated non-infectious uveitis, supporting further evaluation of ICAM-5 as a candidate circulating biomarker and disease-relevant molecular mediator. Comparable integrative strategies have combined public retinal transcriptomics with circulating biomarker validation to identify disease-relevant candidates in retinal disorders (43).

ICAM-5 is traditionally recognized as a neuron-specific adhesion molecule predominantly localized to the soma and dendrites of telencephalic neurons (44). Its core physiological functions center on the regulation of synaptic development and plasticity; notably, ICAM-5 is enriched in dendritic filopodia, where it restrains premature spine maturation and facilitates the formation of functional synapses (45, 46). Structurally, ICAM-5 executes these functions through precise molecular interactions: its extracellular domain binds to β1- and β2-integrins as well as the matrix protein vitronectin, while its cytoplasmic domain associates with α-actinin and ezrin-radixin-moesin (ERM) proteins to modulate cytoskeletal dynamics (47–49). These molecular properties provide a biologically plausible basis for considering ICAM-5 in the context of inflammatory tissue remodeling and neuro-immune interactions.

A central feature of uveitis is the recruitment and infiltration of leukocytes into ocular tissues (50, 51). ICAM-5 is a validated ligand for the β2-integrin lymphocyte function-associated antigen-1 (LFA-1), which is expressed on T cells, macrophages, and other leukocyte populations (44, 52). In this respect, ICAM-5 may share aspects of the adhesive biology of other ICAM family members, particularly ICAM-1, a well-established mediator of leukocyte adhesion and transmigration during inflammation (49). Consistent with this concept, the GSE194060 transcriptomic analysis showed increased *ICAM5* expression in CD1c^+^ conventional type 2 dendritic cells purified from peripheral blood mononuclear cells with non-infectious uveitis (42). Although this finding does not identify the cellular source of circulating ICAM-5, it supports a relationship between ICAM-5 dysregulation and systemic immune activation in non-infectious uveitis.

Another relevant feature of ICAM-5 biology is its proteolytic conversion from a membrane-bound adhesion molecule into soluble ICAM-5 fragments. Under physiological conditions, membrane-bound ICAM-5 can be cleaved by matrix metalloproteinases following glutamate receptor activation, releasing soluble ICAM-5 into the extracellular space and circulation (44, 45). In neurological disorders such as hypoxic-ischemic brain injury and acute encephalitis, increased soluble ICAM-5 levels in serum or cerebrospinal fluid have been associated with neuronal injury and neuroinflammation (53, 54). More recently, serum proteome analysis in systemic juvenile idiopathic arthritis-associated lung disease identified ICAM5, together with MMP7 and allergic/eosinophilic chemokines, as part of a disease-associated protein signature, and immunohistochemistry localized *ICAM5* and MMP7 in affected lung tissue (55). Although this finding comes from a non-ocular inflammatory disease, it supports the broader possibility that circulating ICAM-5 may be associated with inflammatory tissue injury and remodeling beyond classical neuronal contexts. In the context of uveitis, the elevated plasma ICAM-5 observed in our cohort may therefore reflect a combination of altered *ICAM5* expression in immune cells and enhanced shedding from inflamed tissues, rather than a single cellular source. This possibility may also contribute to the more pronounced ICAM-5 elevation observed in VKH disease. VKH disease is characterized by diffuse choroidal and neuroretinal inflammation and may be accompanied by meningeal involvement during the acute phase (30, 31), providing a broader neuro-ocular inflammatory context in which ICAM-5 release could be increased. Thus, the higher ICAM-5 levels observed in VKH may reflect more extensive neuro-ocular tissue involvement and inflammation-associated proteolytic remodeling. Soluble ICAM-5 may further influence inflammatory responses through interactions with leukocyte integrins and adhesion-related immune activation (44, 52, 56), although this possibility requires direct validation in uveitis.

A further, more speculative pathway involves the interaction between ICAM-5 and vitronectin within the ocular microenvironment. Vitronectin is an extracellular matrix-associated protein involved in tissue remodeling and inflammatory responses, and ICAM-5–vitronectin binding has been reported to induce ERM protein phosphorylation and actin cytoskeletal reorganization (48). These processes are relevant to endothelial and epithelial barrier regulation and could, in principle, contribute to blood–ocular barrier dysfunction, a central pathological feature of uveitis (1, 4). This proposed pathway should therefore be regarded as a hypothesis generated from the known molecular properties of ICAM-5 rather than as a mechanism directly demonstrated by the present study.

The Human Protein Atlas is a publicly available database; we queried it using the official human gene symbol *ICAM5* and reviewed the resulting tissue-expression profile. *ICAM5* was classified as brain-enriched and detected in the retina, supporting its retinal relevance and the predominantly neuronal expression pattern observed in our single-cell analyses. In addition, retinal single-cell RNA-sequencing data generated in this study showed higher *Icam5* expression in EAU retinal cells than in normal controls (**Supplementary Figure 6**). Analysis of the published GSE132229 dataset (41) further demonstrated that *Icam5* expression was concentrated in retinal neuronal populations, principally amacrine cells and retinal ganglion cells, and was negligible in the displayed intraretinal immune-cell subsets (**Supplementary Figure 7**). Mean normalized *Icam5* expression in retinal ganglion cells was also higher in *Aire-knockout* than in wild-type retinas. These findings are consistent with predominantly neuronal retinal expression of *Icam5* and provide a biological context in which retinal ICAM-5 may participate in neuron-immune interactions during ocular inflammation. However, they do not establish that the retina is the source of circulating ICAM-5, nor do they by themselves demonstrate a direct pathogenic mechanism. More broadly, disease-relevant crosstalk among retinal microglia, neurons, and glia is increasingly recognized in retinal disorders (57).

The main strength of this study is its integration of Mendelian randomization, transcriptomic analysis, clinical protein quantification, and retinal single-cell profiling, providing convergent evidence across complementary biological levels. Several limitations should be acknowledged. First, the modest clinical sample size limited further stratification by disease stage, inflammatory activity, or duration. Although age imbalance was addressed by covariate adjustment, residual confounding cannot be completely excluded. Second, the genetic analyses were based on European-ancestry GWAS data, whereas the clinical cohort comprised Han Chinese participants, which may limit generalizability to East Asian populations. Third, although *ICAM5* did not meet the prespecified FDR threshold, it showed consistent nominal associations across independent transcriptomic and proteomic resources, supporting its prioritization for downstream evaluation. *ICAM5* should therefore be considered a genetically supported candidate mediator, while its definitive causal role requires further investigation. Finally, the proposed roles of ICAM-5 in immune adhesion, proteolytic shedding, barrier remodeling, and neuron-immune interactions require direct functional validation; larger multi-center cohorts and dedicated mechanistic studies are needed to confirm these findings and clarify the role of ICAM-5 in ocular inflammation.

## 5 Conclusions

Our study integrates multi-omics Mendelian randomization with clinical protein quantification, transcriptomic profiling, and retinal single-cell analyses to evaluate the relevance of ICAM-5 in immune-mediated non-infectious uveitis. Genetically predicted *ICAM5* expression and protein levels showed directionally consistent associations with uveitis susceptibility across the eQTLGen, cis-restricted Icelandic SomaScan, and cis-restricted FinnGen R10 datasets, with no evidence of reverse causation or substantial horizontal pleiotropy. Clinical validation further demonstrated elevated plasma ICAM-5 concentrations across three major uveitis subtypes, with the most pronounced increase observed in VKH disease, and these associations persisted after adjustment for age and sex. Together, these findings support ICAM-5 as a genetically supported candidate mediator and circulating biomarker of immune-mediated non-infectious uveitis. Further studies are needed to clarify its cellular sources, contribution to ocular inflammation, and potential therapeutic relevance.

## Data Availability

The raw retinal single-cell RNA-sequencing data generated in this study have been deposited in the Genome Sequence Archive (GSA) at the National Genomics Data Center (NGDC), accession number CRA050653 (BioProject PRJCA074793; https://ngdc.cncb.ac.cn/gsa/browse/CRA050653). Publicly available datasets analyzed in this study, including GSE194060 and GSE132229, are identified in the Materials and Methods section.
