# Supplementary material for: Genetic susceptibility and clinical validation of ICAM-5 in immune-mediated non-infectious uveitis
Source: Front Immunol. 2026 Oct 6;17:1948697. doi: 10.3389/fimmu.2026.1948697 (PMC13680458; doi:10.3389/fimmu.2026.1948697)
Supplement: Supplementary file 1 [file SupplementaryFile2.docx]

**Supplementary Figure 1.** Venn diagram for overlapping MR candidates across three omics datasets

**Supplementary Figure 2.** Scatter plots of the genetically predicted associations of ICAM5 expression and circulating ICAM-5 abundance with uveitis susceptibility across three molecular QTL datasets

**Supplementary Figure 3.** Reverse Mendelian randomization analysis of the association between genetically predicted uveitis liability and circulating ICAM-5 abundance in the FinnGen R10 and Icelandic pQTL datasets

**Supplementary Figure 4.** Multivariable model for plasma ICAM-5

**Supplementary Figure 5.** *ICAM5* expression in CD1c+ conventional type 2 dendritic cells purified from PBMCs in GSE194060

**Supplementary Figure 6.** Violin plot of scaled *Icam5* expression in NC and EAU mouse retinas profiled by sc-RNAseq(CRA050653)

**Supplementary Figure 7.** *Icam5* expression across retinal cell subsets from WT and AireKO mice in GSE132229
